# Down-Regulated E-Cadherin Expression Is Associated with Poor Five-Year Overall Survival in Bone and Soft Tissue Sarcoma: Results of a Meta-Analysis

**DOI:** 10.1371/journal.pone.0121448

**Published:** 2015-03-30

**Authors:** Ning Wang, Yong-Lai He, Li-Juan Pang, Hong Zou, Chun-Xia Liu, Jin Zhao, Jian-Ming Hu, Wen-Jie Zhang, Yan Qi, Feng Li

**Affiliations:** 1 Department of Pathology, Shihezi University School of Medicine, Shihezi, Xinjiang, China; 2 Key Laboratories for Xinjiang Endemic and Ethnic Diseases, Shihezi University and Chinese Ministry of Education, Shihezi, Xinjiang, China; 3 Department of ICU intensive care, the First Affiliated Hospital, Shihezi University School of Medicine, Shihezi, Xinjiang, China; University of North Carolina School of Medicine, UNITED STATES

## Abstract

**Purpose:**

To conduct a meta-analysis to evaluate the prognostic role of E-cadherin expression in bone and soft tissue sarcomas.

**Methods:**

The PubMed, EMBASE, and Web of Science databases were searched using terms related to E-cadherin, sarcoma, and prognosis for all articles published in English before March 2014. Pooled effect was calculated from the available data to evaluate the association between negative E-cadherin expression and 5-year overall survival and tumor clinicopathological features in sarcoma patients. Pooled odds ratios (OR) and risk ratios (RR) with 95% confidence intervals (CI) were calculated using a fixed-effects model.

**Result:**

Eight studies met the selection criteria and reported on 812 subjects. A total of 496 subjects showed positive E-cadherin expression (59.9%). Negative E-cadherin expression in bone and soft tissue sarcomas was correlated with lower 5-year overall survival (OR = 3.831; 95% CI: 2.246–6.534), and was associated with higher clinical stage (RR = 1.446; 95% CI: 1.030–2.028) and with male sex (RR = 0.678; 95% CI: 0.493–0.933).

**Conclusion:**

In the E-cadherin negative group, 5-year overall survival was significantly worse than in the E-cadherin positive group. However, further studies are required to confirm these results.

## Introduction

Sarcomas are a heterogeneous group of mesenchymal neoplasms that can be grouped into two general categories: soft tissue sarcoma (STS) and primary bone sarcoma (PBS) [1]. There are more than 100 distinct subtypes of sarcomas; STSs include liposarcoma, leiomyosarcoma, synovial sarcoma, rhabdomyosarcoma, and angiosarcoma; PBSs include osteosarcoma, Ewing’s sarcoma, chondrosarcoma, and giant cell tumor. Sarcomas account for less than 1% of all malignancies [2]. Despite recent advances in treatment, sarcoma prognosis varies greatly between subtypes. Traditional therapeutic modalities are used in the treatment of most sarcoma subtypes, which, in many cases, are resistant to adjuvant therapies [3].

E-cadherin (also known as cadherin 1) is a cell-cell adhesion molecule that regulates histogenesis and stabilization and differentiation of the epithelium [4]. However, only normal E-cadherin expression plays an important role in tissue architecture and maintenance of tissue integrity. Down-regulation of E-cadherin expression results in the breaking of cell-cell contacts and the cells become mesenchymal cells [5]. In cancerous cells, cell may transition from an epithelial to a mesenchymal like form (epithelial-to-mesenchymal transition) [6].

It has been confirmed by a number of studies that reduced E-cadherin expression is correlated with many types of carcinomas, including colorectal, breast, and ovarian cancers [7–9]. Although E-cadherin expression has been primarily examined in carcinoma, it has been demonstrated that reduced E-cadherin expression plays a pivotal role in differentiation, invasion, and metastasis in several malignancies, including certain sarcomas [10]. However, there have been no comprehensive and detailed meta-analysis studies of E-cadherin expression in malignant bone and soft tissue sarcomas that evaluate the impact of E-cadherin expression on the constitution of the architecture of these tumors or have determined the potential relationship between E-cadherin and overall survival. Thus, we performed a meta-analysis to examine the association between E-cadherin expression and prognosis in a number of bone and soft tissue sarcomas.

## Methods

### Publication search

We carried out a search of the PubMed, EMBASE, and Web of Science databases using the terms “E-cadherin” or “CDH1” in combination with the terms “sarcoma” or “soft tissue sarcoma” and using terms related to “prognosis,” including “outcome,” “prognosis,” “prognostic,” and “survival” with all possible term combinations. The search was updated most recently on March 24, 2014. To manually identify additional studies, the references of all the studies identified in the databases were explored. The bibliographies of review articles and other pertinent articles were also inspected to identify relevant studies.

### Inclusion and exclusion criteria

In order to be included in this meta-analysis, studies needed to meet specific criteria: (a) sarcoma patients included in the studies were confirmed histopathologically; (b) the association of E-cadherin expression with sarcoma was evaluated; (c) Kaplan–Meier survival analysis or 5-year overall survival was performed or clinicopathological features were evaluated; and (d) full text articles were written in English. Studies were excluded from the meta-analysis if (a) the study was reported in an abstract, letter, review, expert opinion, or case report; (b) the study was a laboratory, animal, or in vitro study; (c) the results overlapped or duplicated previously reported data; or (d) the study did not present information required for survival analysis.

### Data extraction

Data was extracted from all included studies to a standardized form that included the first author’s name, year of publication, number of patients, country of origin, size of tumor, recurrence or metastasis, TNM stage, cut-off value for E-cadherin positivity, and overall survival (OS) curves or 5-year overall survival for patients with positive and negative E-cadherin expression.

### Statistical analysis

The prognostic value of positive E-cadherin expression in sarcoma patients on 5-year overall survival was evaluated, after correcting for sex, age, histological grade, tumor size, distant metastasis, and TNM stage. A number of variables were compared including male patients versus female patients, clinical stages I–II versus clinical stages III–IV, tumors larger than 5 cm in size versus tumors less than 5 cm in size, and patients older than 20 years versus patients younger than 20 years.

The meta-analysis was performed using STATA software (version 12.0). For individual studies, the survival analysis between E-cadherin positive and negative groups was considered to be significant when the P-value was less than 0.05 through univariate analysis (two-tailed test). Dichotomous variables were analyzed through the estimation of risk ratios (RR) or odds ratios (OR) with 95% confidence intervals (CI). The pooled effect was calculated using a fixed-effects model. Heterogeneity between studies was evaluated by the Chi-square test and expressed by *I*2 index, with *I*2 > 50% considered to be heterogeneity.

To identify publication bias, the Egger’s test and contour-enhanced funnel plot were performed. Publication bias was considered to be significant when P < 0.05. In addition, the contour-enhanced funnel plot indicates regions of statistical significance. Causes of asymmetry, such as variable study quality, were assessed through overlay of contour-enhanced funnel plots.

## Results

### Study selection and characteristics

**Fig 1:**
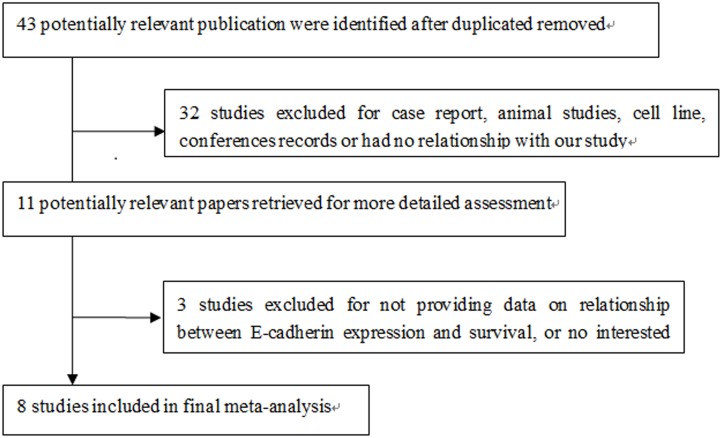
Process of study selection.

In total, 43 potentially relevant citations were identified through the publication search (Fig 1). Two reviewers independently evaluated the titles and abstracts of the 43 potential articles. A total of 28 articles were determined to be irrelevant and 4 articles were identified as review articles or case reports on E-cadherin expression in sarcoma. The remaining 11 articles were read in full, and 8 studies, reporting the results of 812 cases, were included in the meta-analysis.

The main results of the relevant studies were summarized and are shown in Table 1. In the 8 sarcoma studies [11–18], sample sizes ranged from 31 to 378, with a total sample of 812 sarcoma cases. Included in this meta-analysis were 378 Ewing’s sarcomas, 107 osteosarcomas, 167 synovial sarcomas, 86 leiomyosarcomas, and 74 other STSs. A total of 496 sarcoma cases showed positive E-cadherin expression (59.9%).

**Table 1:** Main characteristics of the included studies.

First author (year)	Country of origin	Type of sarcoma	Antibody source	Definition of E-cadherin negative	Sample size (M/F, n)	Mean age	Source dates	Expression rate	5-yr OS rate (%)
Jinyoung Yoo [11] (2001)	South Korea	soft tissue sarcomas	ABD Co, Lexington	No expression	91	NR	1992–1998	12%	NR
Tsuyoshi Saito[12] (2001)	Japan	synovial sarcoma	Transduction	Less than 10% of the cells were stained [19]	31(11/20)	65.9	NR	25.8%	35.5
Teiyu Izumi[13] (2007)	Japan	synovial sarcoma	Transduction	Less than 90% of the cells were stained	92(35/57)	NR	1955–2004	34.8%	56.5
Jilong Yang[14] (2010)	China	leiomyosarcoma	NR	NR	31	NR	NR	58%	58.1
Ke Yin [15] (2012)	China	osteosarcoma	R&D Systems	Summing the intensitys core and expressions core = 0	107(64/43)	NR	2000–2010	20.6%	44.9
Isidro Machado[16] (2012)	Spain	Ewing's sarcoma	Novocastra	Mild in <5%	378	NR	NR	97%	54.9
Wei Tian[17] (2013)	China	leiomyosarcoma	ZM-0092 Zhongshan	Summing the intensitys core and expressions core ≤ 2	45(18/27)	58	NR	15.6%	26.7
Yan Qi[18] (2013)	China	synovial sarcoma	DAKO	less than 1% positive tumor cells	37(18/19)	36	1968–2011	56.8%	33.3

Note: NR, not report; OS, overall survival.

Full survival data was presented for 7 of the studies, and these studies assessed the association of E-cadherin expression with OS for 526 patients (195 Ewing’s sarcomas, 107 osteosarcomas, 148 synovial sarcomas, and 76 leiomyosarcomas). Kaplan–Meier survival analysis was performed in 6 of the studies to determine the association of E-cadherin expression with overall survival. One study presented the data in sufficient detail to calculate overall survival. The follow-up period in 7 of the studies was more than 60 months. The studies were conducted in 3 countries (China, Japan, and Spain).

### Correlation between E-cadherin negative expression and 5-year overall survival

**Fig 2:**
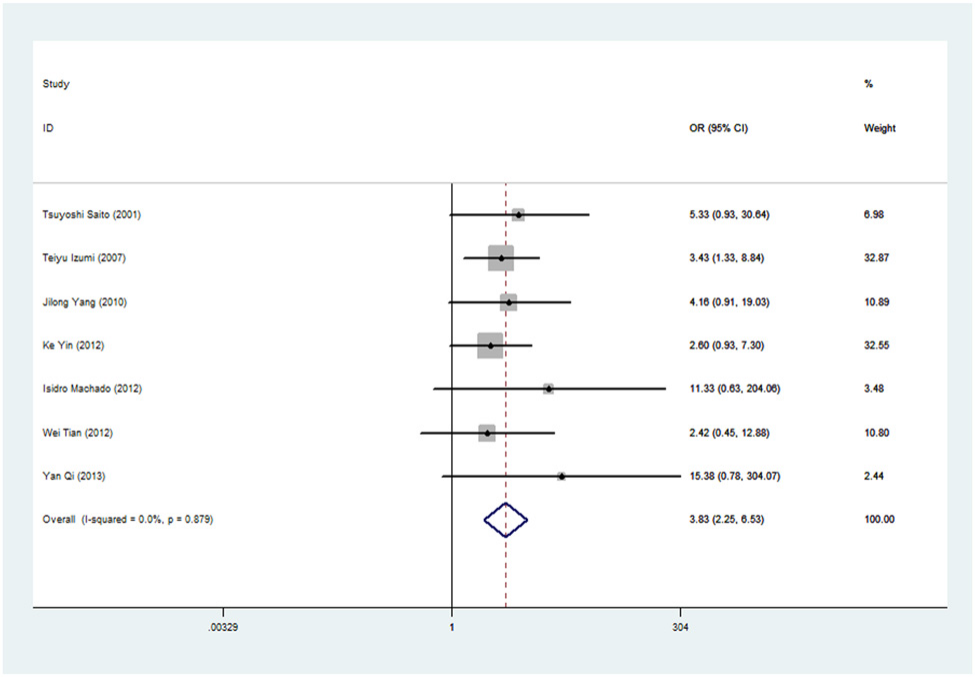
Odd ratios (OR) and 95% confidence intervals (CIs) in 7 studies assessing the relationship between negative E-cadherin expression and overall survival (OS) in patients with sarcomas. (P = 0.000, OR = 3.831; 95%CI: 2.246–6.534). (fixed-effect model analysis).

From the probability graphs of 7 of the studies, 5-year overall survival data were extracted. Meta-analysis, performed using the fixed effects model (I2 = 0.0%; Fig 2), showed that negative E-cadherin expression correlated with a lower 5-year overall survival (OR = 3.831; 95% CI: 2.246–6.534) in sarcomas.

**Fig 3:**
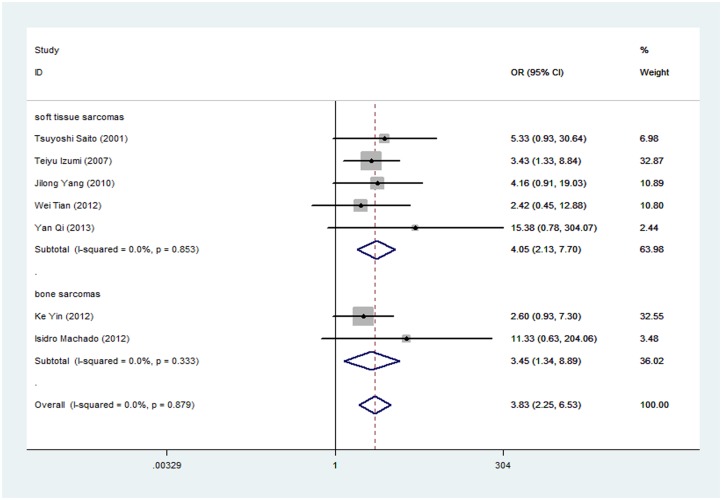
Forest plot (fixed effects model) about the association between E-cadherin expression with overall survival in patients with soft tissue sarcomas and bone sarcomas respectively. (P = 0.000, OR = 4.047; 95%CI: 2.127–7.700) (P = 0.01, OR = 3.447; 95%CI: 1.336–8.893). (fixed-effects model analysis).

Soft tissue sarcomas: Assessment of the association of E-cadherin expression and 5-year overall survival in STS cases resulted in a pooled OR of 4.047 (95% CI: 2.127–7.700; Z = 4.26; P = 0.000; Fig 3), indicating that the loss of E-cadherin was significantly associated with poorer prognosis in STS.

*Bone sarcomas*: Down-regulated expression of E-cadherin correlated with lower 5-year overall survival in bone sarcoma cases. The pooled OR was 3.447 (95% CI: 1.336–8.893; Z = 2.56; P = 0.01; Fig 3), indicating that reduced E-cadherin expression was associated with unfavorable prognosis in PBS.

### Correlation between E-cadherin negative expression and tumor clinicopathological features

**Fig 4:**
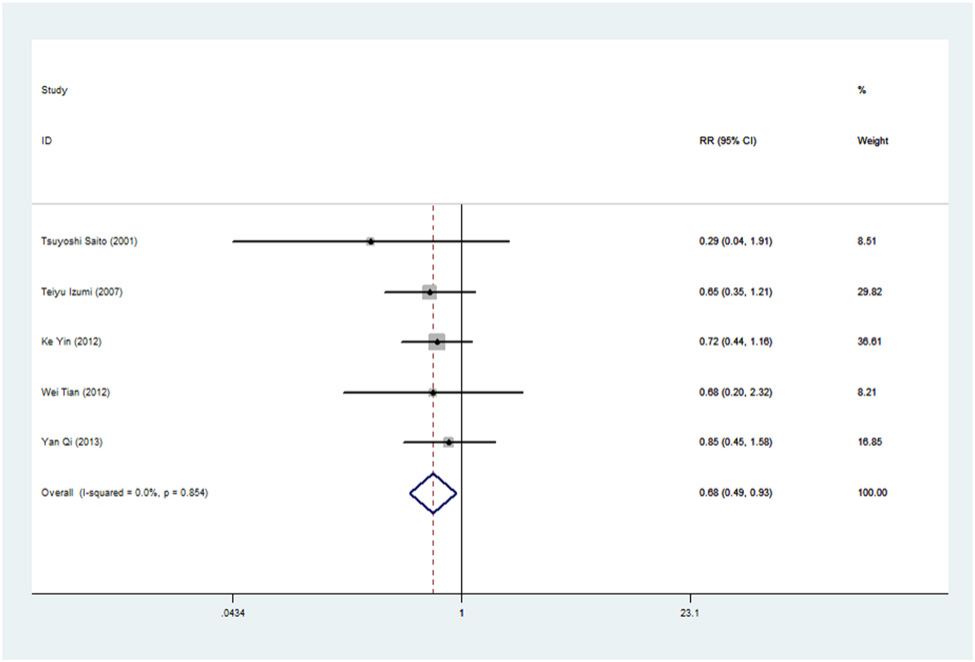
Risk ratio (RR) and 95% confidence intervals (CIs) in studies assessing the relationship between E-cadherin expression and male patients in sarcoma. (P = 0.017, RR = 0.678; 95%CI: 0.493–0.933).

While the E-cadherin protein expression was not associated with patients’ sex in each study, a relationship between E-cadherin expression and sex of sarcoma patients was reported in 5 studies. Interestingly, the pooled data from these 5 studies showed that E-cadherin expression correlated significantly with sex (RR: 0.678; 95% CI: 0.493–0.933; P = 0.017; Fig 4). Thus, reduced E-cadherin expression occurred more often in male sarcoma patients.

**Fig 5:**
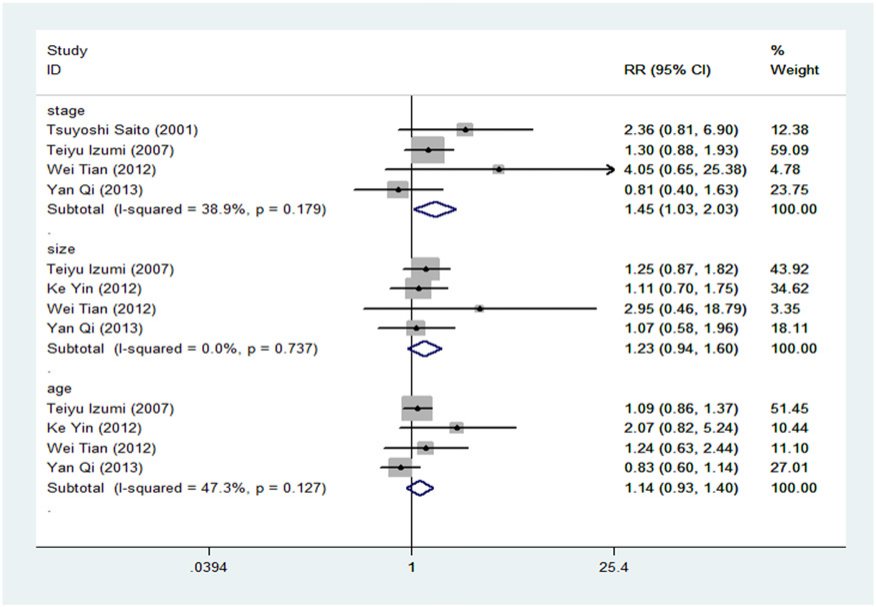
Risk ratios (RRs) and 95% confidence intervals (CIs) in studies assessing the relationship between E-cadherin negative expression and the higher stage (III–IV). (RR: 1.446, 95% CI: 1.030–2.028, P = 0.033); large tumor (>5cm) (RR: 1.226, 95% CI: 0.941–1.597, P = 0.130); and old age (>20 year-old) (RR: 1.138, 95% CI: 0.927–1.398, P = 0.216).

Four studies assessed the correlation of E-cadherin expression with clinical stage. The pooled RR was 1.446 (95% CI: 1.030–2.028; Z = 2.13; P = 0.033; Fig 5), indicating that low E-cadherin expression was associated with higher clinical stages (III–IV). The association between E-cadherin expression with tumor size and patient age in sarcomas was presented in 4 studies. However, E-cadherin expression did not significantly correlate with tumor size (P = 0.130) or patient age (P = 0.216; Fig 5).

### Publication bias

**Fig 6:**
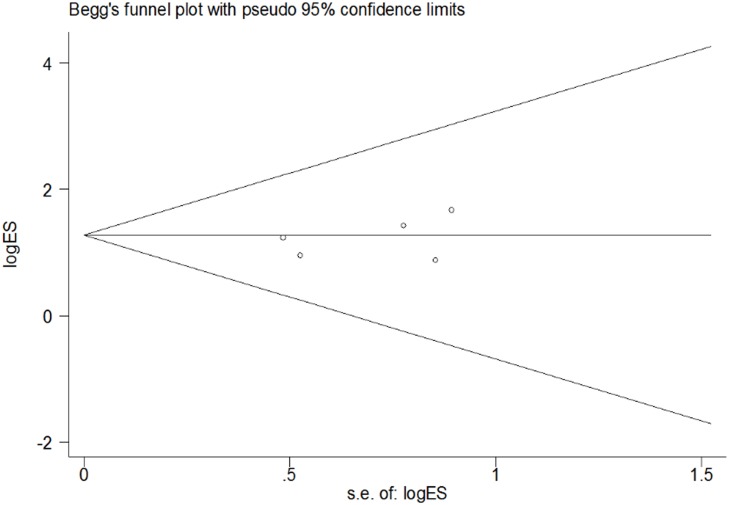
Funnel plots of Begg’s were used to Evaluate Publication Bias on odd ratios. Studies is symmetrically distributed which suggest lack of publication bias.

Publication bias was assessed using contour-enhanced funnel plots [20] and Egger’s test [21] and was used to evaluate the reliability of the results of the meta-analysis, especially for the associations that showed statistical significance. The shapes of the funnel plots showed that no publication bias was observed (Fig 6). Furthermore, we used influence analysis to evaluate the influence of a single study on the summary effect. The meta-analysis was not dominated by any individual study, and the removal of any study did not affect the observed associations.

## Discussion

To our knowledge, this study is the first comprehensive and detailed meta-analysis to assess the association of down-regulated E-cadherin expression with 5-year overall survival and tumor clinicopathological features in sarcoma patients. Our results provide a foundation and new target for clinical decision-making in the diagnosis and treatment of sarcoma.

Through the mediation of cell-cell adhesion, E-cadherin plays a critical role in tissue architecture and the maintenance of tissue integrity, controlling the growth and development of cells [22–24]. The down-regulation of E-cadherin is associated with the process known as epithelial to mesenchymal transition (EMT), which leads to increased invasion and metastasis during tumor progression in multiple carcinomas of epithelial origin [4]. The mechanism of EMT in carcinoma suppression has been elucidated [4,25–27]. In contrast, in sarcomas, the expression of E-cadherin is associated with a similar, but reverse, process known as mesenchymal to epithelial reverting transition (MET), which may play an important role in tumors of mesenchymal origin. MET has been observed and reported in some types of sarcoma, including leiomyosarcoma and synovial sarcoma [14,19]; however, the mechanisms of MET in sarcomas remains to be elucidated.

In this meta-analysis, we found that 59.9% of the sarcomas showed positive E-cadherin expression. In addition, up-regulated E-cadherin expression in sarcoma cells was associated with more favorable outcomes than in sarcomas with down-regulated E-cadherin expression. A similar association between reduced E-cadherin expression and carcinoma, including colorectal carcinoma, ovarian cancer, and bladder cancer, has been established. Through meta-analysis of the prognostic significance of E-cadherin in several cancers, including squamous cell carcinoma, colorectal carcinoma, and ovarian cancer [28–30], it has been demonstrated that negative E-cadherin expression is associated with poorer prognosis in carcinoma.

From our meta-analysis, we showed that the absence of E-cadherin expression was significantly correlated with 5-year overall survival, with higher clinical stage (III–IV), and with male sex. STS and PBS are staged and treated through different approaches. This meta-analysis was performed to estimate the association of E-cadherin expression with 5-year overall survival in the two groups and results showed that down-regulated E-cadherin expression was associated with poorer prognosis in both PBS and STS. And indicating that low or absent E-cadherin expression was a valuable prognostic factor in the two general types (STS and PBS). From the studies included in this meta-analysis, we were unable to determine whether E-cadherin expression in sarcoma was associated with any additional clinicopathological features, such as differentiation, recurrence, metastasis or grade; however, it has been established previously that the absence of E-cadherin expression in sarcomas is significantly correlated with poorer differentiation [17], higher grade [13] and earlier metastasis [31] in sarcomas.

Sato *et al* have shown that E-cadherin is expressed in certain sarcomas, especially in those with epithelioid features, such as rhabdomyosarcoma, synovial sarcoma, osteosarcoma, leiomyosarcoma, Ewing’s sarcoma, and clear cell sarcoma [32]. We showed E-cadherin expression in 100% of clear cell sarcoma cases, 31.4% of synovial sarcoma cases, 20.6% of osteosarcoma cases, and 3% of Ewing’s sarcoma cases. Interestingly, most of these sarcomas (clear cell sarcoma, synovial sarcoma, and Ewing’s sarcoma) are all translocation-associated sarcomas [33]. Thus, there may be a link between translocation and the expression of E-cadherin.

In our meta-analysis, the PBSs that were included were osteosarcoma and Ewing’s sarcoma. These two PBSs are the most common primary malignant bone tumors of childhood [34,35]. Ewing’s sarcoma develops most commonly in bone in adolescents, although it can also occur in soft tissue and arise at any age. In addition, Ewing’s sarcoma, unlike osteosarcoma, is responsive to radiation therapy.

There is heterogeneity in the expression of E-cadherin in leiomyosarcoma. While Pinelopi *et al* and Sato *et al* have reported negative E-cadherin expression in leiomyosarcoma cases [10,32], positive E-cadherin expression has also been reported [14,17]. While our results are in agreement with the latter, the observed differences may be a result of the small sample sizes and different cutoff level. For example, Pinelopi *et al* set the cutoff level for positivity at 20% cell staining, which is much higher than the cutoff level of 2% set by Tian *et al*. In our meta-analysis, all liposarcoma cases showed negative E-cadherin expression. The absence of E-cadherin expression likely reflects the mesenchymal origin of liposarcomas and indicates the lack of epithelial differentiation in these tumors [32,36]. Similarly, a lack of E-cadherin expression has been observed in both small (10 patients) and large (71 patients) studies of liposarcomas [32,36]. In epithelioid sarcoma, lack of E-cadherin expression has previously been observed [37,38]. In contrast, we observed that in biphasic synovial sarcoma there was a high rate of positive E-cadherin expression, suggesting a strong relationship between increased E-cadherin expression and presence of glandularity.

Combined, these results suggest that E-cadherin may not function as a tumor suppressor gene only in epithelial cells but also in mesenchymal tumors. However, the absence of E-cadherin expression in normal mesenchymal cells is known to be a differentiation event.

There are some limitations to this meta-analysis. First, the inclusion criteria for the meta-analysis prevented the inclusion of articles published in languages other than English. By excluding articles written in languages other than English, it is possible that study results have not been included in the meta-analysis. In addition, studies that obtain negative or null results are often not published and thus cannot be identified by literature search and cannot be included in the meta-analysis. In addition, reports that did not provide sufficient data were excluded from our analysis, which may have resulted in publication bias. Second, in the studies that were included, the protein assessment methods were inconsistent (including antibody types and concentrations and cutoff value definitions), which could contribute to heterogeneity.

In conclusion, our results suggest that 5-year overall survival in sarcoma cases with negative E-cadherin expression is worse than in sarcoma cases with positive E-cadherin expression, particularly in synovial sarcoma, osteosarcoma and leiomyosarcoma cases. Thus, E-cadherin could become a novel target for strategies aimed at predicting tumor progression and prognosis in sarcoma.

## Supporting Information

S1 PRISMA Checklist PRISMA 2009 Checklist. (DOC)
